# A Case of a Gastrointestinal Stromal Tumor Diagnosed at the Postpartum Period

**DOI:** 10.1155/2016/3621802

**Published:** 2016-11-10

**Authors:** Sefa Kurt, Aras Emre Canda, Emre Karadeniz, Tugba Yavuzsen, Ozgul Sagol, Funda Obuz, Mehmet Serefettin Canda

**Affiliations:** ^1^Dokuz Eylul University School of Medicine, Department of Obstetrics and Gynecology, Izmir, Turkey; ^2^Dokuz Eylul University School of Medicine, Department of Surgery, Izmir, Turkey; ^3^Dokuz Eylul University School of Medicine, Department of Medical Oncology, Izmir, Turkey; ^4^Dokuz Eylul University School of Medicine, Department of Pathology, Izmir, Turkey; ^5^Dokuz Eylul University School of Medicine, Department of Radiodiagnostics, Izmir, Turkey

## Abstract

*Introduction*. We discuss a rare gastrointestinal stromal tumor (GIST) case detected at the 10th postpartum week and we want to pay attention to the challenges and improvements in the diagnosis, surgery, chemotherapy, and follow-up of this rare tumor accompanied with the review of the current literature.* Case Presentation*. A 32-year-old multiparous woman presented with abdominal swelling 10 weeks after her second vaginal birth. Abdominal examination revealed a mass starting from the pelvic level and extending to the right upper quadrant. Radiological examinations showed a solid, multiloculated, and hypervascular mass starting from the pelvis and extending to the transverse colon.* En bloc* mass with a 20 cm jejunal segment resection and a left pelvic side wall peritonectomy with omentectomy was performed. The pathologic examination revealed a high-risk GIST which originated from the jejunum and disseminated to the peritoneum. The patient has been given imatinib 400 mg/day since then. She did not reveal any progression during the 15-month follow-up postoperatively.* Conclusion*. GIST tumors are rare and there is not sufficient information in the literature regarding its management. In this patient having high risk GIST and GIST sarcomatosis we successfully treated the patient by surgery and adjuvant imatinib chemotherapy.

## 1. Introduction

Cancers seen in the reproductive period are the second cause of death after cardiovascular diseases [1]. Although the relative incidence of cancer in pregnancy is identified as 1 in 1000–1500 pregnancies (literature), the upward trend has been observed depending on delaying pregnancy to older ages. In this context, gestational cancer or pregnancy-associated cancer concept is gaining importance nowadays. Pregnancy-associated cancers (PACs) are malignancies diagnosed during pregnancy or in the first year after birth [1]. Breast and cervix cancers take the first place among PAC [2]. GISTs are rare tumors that originated from Cajal cells of gastrointestinal system and c-kit mutation of their progenitor cells [3–6]. GIST is much less common in terms of the definition of PAC. However, in the treatment of GIST, good results with surgical intervention in addition to drugs are received currently and at present, there are significant developments found in this area. A GIST case detected at the 10th postpartum week is presented because of its rarity in pregnancy and the current improvements in its treatment.

## 2. Case Report

**Figure 1:**
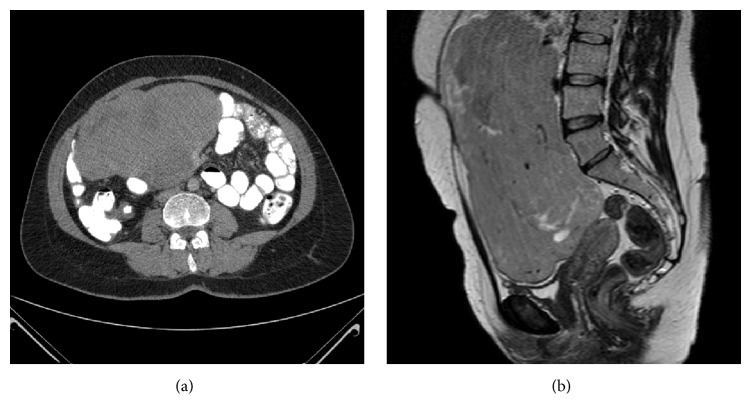
Computerized tomography (a) and magnetic resonance imaging (b) showing a solid, multiloculated, and hypervascular mass starting from the pelvis and extending to the transverse colon, 26 × 22 × 16 cm in size.

A 32-year-old multiparous woman (G3P2) presented with abdominal swelling 10 weeks after her second vaginal birth. She had a normal pregnancy follow-up and birth process, until now, and her medical and family history did not show any feature. In the gynecological examination, external and internal genital organs were normal in size and structure. Postpartum uterine involution was regular. In the abdominal examination, a mass starting from the pelvic level, but with indistinct relation to genital organs and extending to the right upper quadrant, was palpated. In the abdominal ultrasound screening, a multiloculated mass divided with septal structures, including cystic and solid areas starting from the suprauterine level reaching to the subxiphoid region, was detected. In the further evaluation using computerized tomography (Figure 1(a)) and magnetic resonance imaging (Figure 1(b)) a mass starting from the pelvis and extending to the transverse colon, 26 × 22 × 16 cm in size, solid, loculated, hypervascular, and in certain regions associated with some colon segments was observed. CT angiography was performed in order to evaluate the resectability of the mass showed invasion into the superior mesenteric artery and distal branches. Biochemical tests and tumor markers were not diagnostic. According to these findings, a general surgery consultation was requested with the prediagnosis of extragenital tumoral mass.

**Figure 2:**
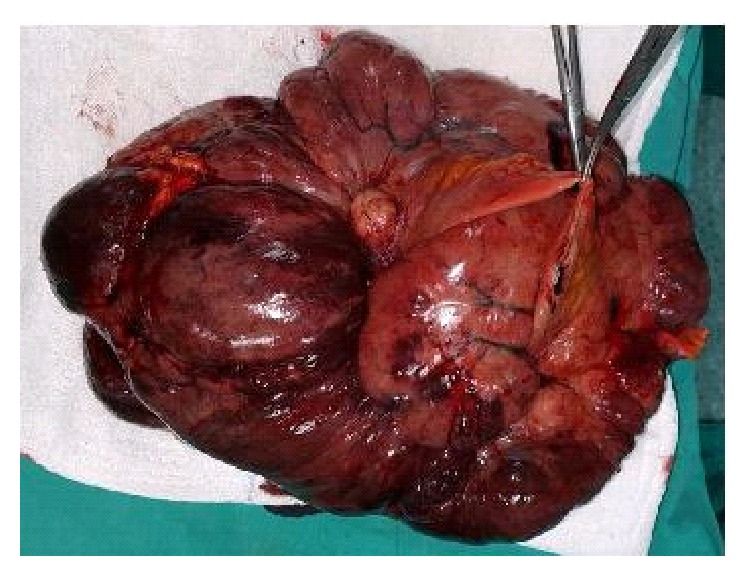
*En bloc *resection specimen: large mass with a 20 cm jejunal segment (surgical clamps holding the proximal and distal stapled ends) left pelvic side wall peritoneum and omentum resection.

Intraoperative observation showed an association between the mass and an approximately 15 cm of jejunal segment; furthermore many millimetric implants were diagnosed on the peritoneum.* En bloc *mass with a 20 cm jejunal segment resection and a left pelvic side wall peritonectomy with omentectomy was performed (Figure 2). Additionally, peritoneal implants were sampled. Frozen section examination was reported as malign stromal tumor. The postoperative period was uneventful and the patient was discharged on the postoperative 7th day.

*Pathologic Examination*. Three peritoneal biopsies (measuring 1 cm to 1.2 cm) were evaluated on frozen section. A spindle cell tumor was seen on microscopy but the surgeon was informed that the subtype of the tumor could be given on paraffin sections. Small intestinal resection together with resection of a large lobulated mass, originating from the serosal surface and mesentery of the intestine, measuring 27 × 22 × 15 cm and weighing 2991 gr, and omentectomy specimen were also submitted. On cross section, the tumor was lobulated, hemorrhagic, soft, and friable with necrotic areas. On microscopy, spindle cell tumor with infiltrative pattern of growth, identical with the tumors seen on peritoneal surfaces, was seen. On immunohistochemistry, tumor cells with moderate atypia were strongly and diffusely positive with C-kit, CD34, and vimentin, focally positive with smooth muscle actin and caldesmon, and negative with S-100, inhibin, desmin, and keratin. Ki-67 index was <5% when counted on 2000 cells. Six mitoses were counted per 50 high power fields or 5 mm^2^. Omentum did not contain any tumor.

The tumor was reported as GIST with peritoneal dissemination and as a high-risk group tumor according to Miettinen and Lasota [7], because of its large size (27 × 22 × 15 cm) and >5 mitotic activity (6/5 mm^2^). Molecular mutational analysis was not performed.

The patient was given imatinib 400 mg/day and is still continuing. She did not reveal any progression during the 15-month follow-up postoperatively and during the writing of this manuscript.

## 3. Discussion

Lack of literature-based evidence and being relatively less common are such difficulties associated with the management of PAC [1]. A malignancy complicating pregnancy creates a dramatic situation in many respects and shows mainly the following characteristics [2]: (a) the overlapping symptoms of malignancy and pregnancy symptoms, like nausea, vomiting, breast changes, abdominal pain, and so forth; (b) changes that occur in the breast and uterus during pregnancy masking the malignity symptoms; (c) the limitations of using imaging and laboratory methods in the diagnosis of malignancy. Moreover, regarding the use of antineoplastic therapy, the limitation of the large prospective studies is a major challenge for the clinician. The clinician faces the difficulty of treating the disease because of poor evidence based on case reports, retrospective small studies, and individual experience. And also, the benefits and risks of malignancy diagnosis and treatment during pregnancy require a very sensitive and a sophisticated balance both for the mother and the baby [2].

The process of the management of a diagnosed malignancy after the birth may lead to many negative consequences related to the breastfeeding, infant, and maternity care, although the risk related to the fetus is reduced. In our patient, breastfeeding is stopped because of delicate maternity process which is interrupted due to surgical intervention, healing process, and adjuvant chemotherapy treatment. The survival rates after the treatment of cancers related or not related to PAC are reported to be not significantly different [1, 8, 9]. However, in some cancers like breast, ovary, and malignant melanoma that are diagnosed during pregnancy or lactation period, the survival rates are reported to be worse than cancers not associated with PAC [8, 9]. Hormonal changes associated with pregnancy, immunosuppression, and increased vascularity have been proposed as factors [10]. This situation for pregnancies after cancer is defined as healthy mother effect [8, 9]. The most common malignancies among PAC are breast, cervix, melanoma, thyroid, and Hodgkin's lymphoma while less common ones include leukemia, ovarian, lung, and gastrointestinal malignancies [11]. The incidence of PAC is not different from nonpregnant women in the same age group. The incidence of colorectal cancer in pregnancy is 1 in 13,000 pregnancies [11]. Diagnosis is often delayed because of pregnancy despite the presence of symptoms such as pain, constipation, and rectal bleeding. The incidence of GIST is 2 in 100,000 with male dominance and reaches to its peak between 5th and 7th decades [12, 13]. Considering these aspects, coming up in a 32-year-old woman, at the 10th postpartum week with a large abdominal mass is an unexpected dramatic situation.

Due to the limited number of cases, in which direction the healthy mother effect would be in GIST is unpredictable. These nonepithelial tumors, which may be localized all over the gastrointestinal system, emerge from the interstitial cells of Cajal in the myenteric plexus or its precursors. Therefore, GIST is also called Cajal tumors [13]. According to its location GIST can present with many different symptoms. According to tumor size and location, it can lead to symptoms such as bleeding, pain, loss of appetite, difficulty in swallowing, ileus, or perforation [12]. It also may be detected incidentally. The biological behavior of GIST in which the diagnosis and treatment significantly improved in recent years shows a wide range from benign cases towards malignant aggressive disease [10]. The most important prognostic factors are tumor size, mitotic index, the origin of the tumor, and C-kıt mutations. Tumors that are larger than 5 cm and having a mitotic index >5 tend to be more aggressive. The presence of C-kit mutations, intestinal origin, and the presence of peritoneal metastasis are poor prognostic factors [13–15]. Our case had a poor prognosis in terms of tumor location, size, mitotic index (6/50), presence of peritoneal metastasis, and diffuse C-kit involvement.

GIST's main treatment is surgery [15]. After averagely 18–24 months following complete surgical resection, recurrence develops in 50% of the cases. Relapses frequently occur in the liver and peritoneum which are often multifocal. Therefore, adjuvant treatment requirements are questionable. Response to conventional chemotherapy and radiotherapy are low, but they are widely used in order to provide palliation [16]. Imatinib mesylate, a C-kit receptor and tyrosine kinase original inhibitor, is a molecular targeted drug and it is the most important non-surgical treatment alternative. Its efficiency has been proven to use as a neoadjuvant in patients not suitable for surgery and as an adjuvant in patients with poor prognosis [16, 17]. In addition, from this group sunitinib and nilotinib are treatment options for patients who are resistant to imatinib treatment or who cannot tolerate it [17]. Imatinib use during breastfeeding is contraindicated [12, 16]. In our case, we stopped breastfeeding after surgery, before adjuvant imatinib mesylate treatment accompanied with cabergoline. Our patient is on 400 mg/day of imatinib mesylate treatment after the surgery and she did not show any sign of disease progression 15 months postoperatively. Murphy et al. reported that in their community-based research of patients with the diagnosis of GIST also showed predisposition against other malignancies [18]. Among these cancers the most common ones are genitourinary, breast, respiratory, and hematologic malignancies. Therefore, follow-up of these patients after treatment should be done with caution in terms of other system malignancies.

## 4. Conclusion

Depending on the conditions in the developing world, postponing the age of pregnancy is increasing the incidence of cancer seen in pregnancy. PAC diagnosis requires awareness in terms of treatment and follow-up. Basic difficulties in PAC result from the delays in treatment of the mother and worsening of the survival, besides the concerns due to the protection of the vulnerable fetus. Compared with other PACs, GISTs are fairly rare. In numerous terms, our case is followed up without any progression after optimal surgery and adjuvant chemotherapy. In the literature search, we have not come up with a GIST related to PAC; therefore, clinical follow-up will show in which direction the healthy mother effect takes place.
